# Transcriptomic analysis of the mechanisms of alleviating renal interstitial fibrosis using the traditional Chinese medicine Kangxianling in a rat model

**DOI:** 10.1038/s41598-020-67690-3

**Published:** 2020-06-30

**Authors:** Yufeng Jiang, Yaohan Zhu, Timing Zhen, Jie Li, Kaichen Xing, Liqun He, Sibo Zhu

**Affiliations:** 1 Department of Nephrology, Shuguang Hospital, Affiliated to Shanghai University of Traditional Chinese Medicine, 185 Puan Road, Shanghai, 200021 People’s Republic of China; 2 Key Laboratory of Liver and Kidney Diseases (Shanghai University of Traditional Chinese Medicine), Ministry of Education, Shanghai, People’s Republic of China; 3 School of Life Sciences, Fudan University, 2005 Songhu Road, Shanghai, 200438 People’s Republic of China; 4 Shanghai Cinoasia Institute, Shanghai, 200438 People’s Republic of China

**Keywords:** Drug discovery, Molecular biology

## Abstract

Renal interstitial fibrosis (RIF) is currently recognized as a crucial mechanism of the pathogenesis of chronic kidney disease (CKD). Kangxianling (KXL, anti-fibrin) is a traditional Chinese medicine that has been proven to significantly reduce the levels of ECM deposition and inhibit renal fibrosis. To characterize the mechanisms and drug targets of KXL, we established a RIF rat model and treated the rats with KXL and losartan. Histological analyses validated the establishment of the RIF model and the treatment effect of KXL. Multiple levels of transcriptomic datasets were generated using lncRNA, mRNA and microRNA sequencing of kidney tissues. Functional annotations and pathway analyses were performed to unravel the therapeutic mechanisms. A multi-level transcriptomic regulatory network was built to illustrate the core factors in fibrosis pathogenesis and therapeutic regulation. KXL and losartan significantly reduced the progression of RIF, and a better therapeutic effect was shown with higher concentrations of KXL. According to the cluster analysis results of the RNA-seq data, the normal control (NC) and high concentration of KXL (HK) treatment groups were the closest in terms of differentially expressed genes. The WNT, TGF-β and MAPK pathways were enriched and dominated the pathogenesis and therapy of RIF. miR-15b, miR-21, and miR-6216 were upregulated and miR-107 was downregulated in the fibrosis model. These small RNAs were shown to play critical roles in the regulation of the above fibrosis-related genes and could be inhibited by KXL treatment. Finally, based on the lncRNA datasets, we constructed a mRNA-lncRNA-miRNA coexpression ceRNA network, which identified key regulatory factors in the pathogenesis of kidney fibrosis and therapeutic mechanisms of KXL. Our work revealed the potential mechanism of the Chinese medicine Kangxianling in inhibiting renal interstitial fibrosis and supported the clinical use of KXL in the treatment of kidney fibrosis.

## Introduction

Chronic kidney disease (CKD) is a chronic disease characterized by the gradual and irreversible loss of renal function over months or years. The incidence of CKD has increased globally with the prolongation of human life expectancy and increased incidence of diabetes and hypertension. A 2012 study found that the incidence of CKD in the Chinese population was 10.8%^1,2^, and according to the WHO (World Health Organization), the number of people that died from CKD in 2012 was 864,226 (mortality rate of 1.5%), which has become a heavy burden on public health resources^3,4^.

Renal interstitial fibrosis (RIF) and glomerulosclerosis are currently considered important pathophysiological mechanisms in the pathogenesis and progression of CKD^5,6^. In addition, it has been suggested that there is a close correlation between the degree of RIF and glomerular sclerosis with renal function^7,8^.

The mechanism of RIF formation is not fully understood at present, but it is generally believed to be associated with dysregulated synthesis of the extracellular matrix (ECM). Recent studies have also shown that epithelial-mesenchymal transition (EMT) is one of the important mechanisms of RIF formation^5^. Renal tubular fibrosis is a pathological process that is induced by cytokine overexpression, dysregulation of renal interstitial cells through secretion of intercellular matrix, and destruction of renal tissue structure, which ultimately lead to kidney failure.

Cytokines are key factors in the progression of renal fibrosis and control the transcription of renal fibrosis-related genes through various signalling pathways. The renal fibrosis signalling pathway involves the transforming growth factor-β/Smad transduction (TGF-β/Smads) signalling pathway, mitogen-activated protein kinase cascade (MAPK) signalling pathway, and adenosine signalling pathway^9,10^. Among them, the relationship between TGF-β/Smads and renal fibrosis is the most important. TGF-β mainly inhibits Smad7 through its downstream signalling molecules Smad2/3 to regulate renal fibrosis^11^. Studies have also implied that the WNT signalling pathway is involved in tissue damage and plays an important role in the progression of renal fibrosis^12^.

In anti-renal fibrosis treatment using traditional Chinese medicine, the core therapeutic strategy is to reconstruct the microenvironment of the kidney^13^. Studies revealed that Huoxue Huayu Decoction showed an anti-fibrotic effect by upregulating the nitric oxide synthase and thus upregulating the nitric oxide content, further causing downregulation of TGF-β1 expression and reducing renal interstitial fibrosis. This pathway was shown to delay the pathogenesis of glomerular sclerosis, providing a protective effect on the kidneys^14^. Kangxianling (KXL, anti-fibrin) is a traditional Chinese herbal formula that can significantly reduce the levels of serum creatine, total cholesterol, and ECM deposition and inhibit renal fibrosis^15^. In previous clinical studies, KXL significantly decreased serum creatinine (Scr) and blood urea nitrogen (BUN) levels as well as delayed the progression of chronic renal failure. KXL significantly lowered creatinine and urea nitrogen levels in early metaphase chronic renal failure (CKD2-3) patients and was widely used in the treatment of early metaphase chronic renal failure^13^.

Based on the above bibliographic research, we postulated that the anti-fibrotic effect of KXL was regulated by changes in the transcriptome. However, the mechanisms and drug targets of KXL are still unknown. Therefore, we built a kidney fibrosis model with KXL and losartan treatment to provide new insights into the anti-fibrotic molecular function of KXL.

## Materials and methods

### Renal fibrosis rat model

**Figure 1:**
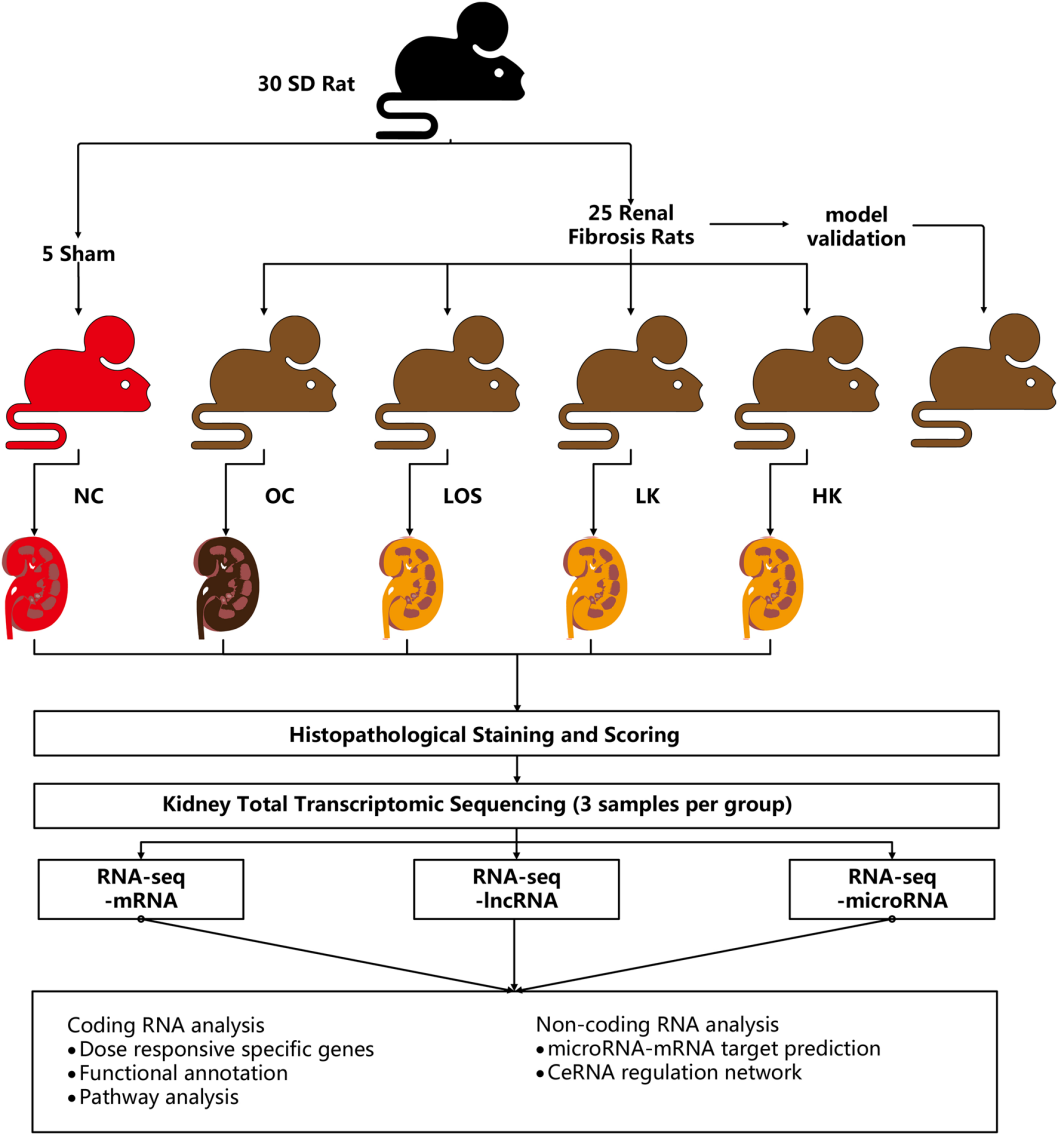
Experimental design and analytical procedures. SD rats were divided into a control group and a RIF model group. The model rats were further divided into 5 groups (n = 5 per group): model validation group, OC group (RIF sham control), HK group (high concentration of KXL), LK group (low concentration of KXL) and LOS group (losartan treatment). The model validation group rats were subjected to renal histopathological section staining. The NC group (sham control) received sham and empty vehicle treatment. Histological analyses validated the establishment of RIF modelling and the treatment effect of KXL. Multi-level transcriptomic datasets were generated using lncRNA, mRNA and microRNA sequencing of kidney tissues. Functional annotations and pathway analyses were applied to unravel the therapeutic mechanisms. A multi-level transcriptomic regulatory network was built to illustrate the core factors in fibrosis pathogenesis and therapeutic regulation.

The study was approved by the Animal Research Ethics Committee of the Shanghai University of Traditional Chinese Medicine (Ethical permit number: SZY-201601007). Animal handling and procedures used in this study were in accordance with the guidelines of the Animal Research Ethics Committee of the Shanghai University of Traditional Chinese Medicine. Thirty SD rats (male, 8 weeks, weight 200 g) were divided into two groups: the NC group (normal control, n = 5) and the model group (n = 25) according to the protocol by Goncalves et al.^16^. Briefly, the right kidneys of the model group were renal ablated. After one week, doxorubicin (ADR) was injected into the tail vein at 5 mg/kg, and the injection was repeated once every 3 weeks at 3 mg/kg to construct a renal fibrosis model. Before the treatment experiment (8 weeks after operation), the model validation group rats were subjected to renal histopathological section staining. Renal pathology showed glomerular mesangial proliferation, increased matrix, tubular atrophy, and interstitial fibrosis, suggesting successful modelling. The NC group rats underwent anaesthesia, ventral laparotomy, and manipulation of the renal pedicles, without any removal of renal mass or ADR injection. Experimental design and analytical procedures were shown in Fig. 1.

### Preparation of KXL decoction

KXL is composed of 15 g radix *Salvia miltiorrhizae*, 15 g *Fructus jujubae*, 15 g radix *Achyranthis bidentatae*, 15 g semen *Prunus persicae*, and 15 g shorthorned Epimedium. All herbal drugs were prepared at the Shuguang Hospital affiliated with Shanghai University of Chinese Medicine, and the decoction was adjusted to 1.5 g/ml with water for the low dose and 4.5 g/mL for the high dose. Losartan (LOS) was purchased from Merck & Co., Inc (New Jersey, USA).

### KXL and LOS treatment

SD rats were divided into a control group and a RIF model group. The control group (NC, sham control) underwent sham operation and received gavage with normal saline. The model group was divided into 4 groups (n = 5 per group): OC group (RIF sham operation control, gavage with normal saline); HK group (high concentration of KXL at a daily dose of 1.5 mL (4.5 g/mL) per 100 g body weight); LK group (low concentration of KXL, 1.5 mL (1.5 g/mL) per 100 g body weight) and LOS group (losartan-treated group, 40 mg per 100 g body weight). After gavage for 8 weeks, the levels of Scr, BUN and 24 h urine protein excretion were determined. The animals were sacrificed, and the left kidneys were collected in paraformaldehyde for pathological staining and in TRIzol (Invitrogen, USA) for RNA-seq studies. Haematoxylin–eosin (HE) and Masson staining were performed using the Haematoxylin and Eosin Staining Kit (C0105, Beyotime, Shanghai, China) and Masson’s Trichrome Stain Kit (G1340, Solarbio). The glomerular sclerosis index (GSI) was scored according to the method by Paij et al*.*^17^. Briefly, 40 glomeruli in each rat were graded according to the following scale: 0, normal; 1, < 25%; 2, 25–50%; 3, 50–75%, and 4, > 75% cross-sectional sclerosis. The GSI was calculated for each animal with the following formula: (N1 × 1 + N2 × 2 + N3 × 3 + N4 × 4)/40, where N1, N2, N3, and N4 represent the numbers of glomeruli graded 1, 2, 3, and 4, respectively. The tubulointerstitial injury index (TII) assessment and scoring method were obtained as per the approach by Bian et al.^18^. The TII was observed and graded as follows: grade 0, no morphological deformities; grade 1, < 10%; grade 2, < 25%; grade 3, < 50%; grade 4, < 75%, and grade 5, 75% and higher. The blue stained area in the interstitium was counted as interstitial fibrosis.

### RNA extraction and RT-qPCR

RNA from three out of five kidneys was further purified using a TRIzol kit. The quality of the extracted RNA was evaluated using an Agilent 2100 Bioanalyzer with RNA integrity values (RINs) higher than 8. The primers were designed using Primer Premier 5.0 software (Premier Biosoft International, Palo Alto, CA, USA) and synthesized by Generay Biotech Co., Ltd. (Table 1). RT-qPCR was performed using the KAPA SYBR Green Supermix PCR kit with the AriaMx Real-Time PCR System (both Agilent Technologies, Inc., Santa Clara, CA, USA). The relative expression levels of the different genes were determined using the 2^-ΔΔCT^ method^19^. GAPDH was used for qPCR normalization.

**Table 1:** The sequences of the primers.

Primer	Sequence	Amplicon length (bp)
Rattus-TGFβ1-F	CGCAACAACGCAATCTATGACA	204
Rattus-TGFβ1-R	ACCAAGGTAACGCCAGGAATT	
Rattus-Smad3-F	GAGGAGAAGTGGTGCGAGAAG	197
Rattus-Smad3-R	CACAAGCGGCAGTAGATGACA	
Rattus-Smad7-F	GAGGCTGTGTTGCTGTGAAT	243
Rattus-Smad7-R	CCAGGCTCCAGAAGAAGTTG	
Rattus-Col1a1-F	AGAATATGTATCACCAGACGCAGAA	269
Rattus-Col1a1-R	GACCACGAGGACCAGAAGGA	
Rattus-Col1a2-F	GGCAACAGCAGATTCACCTACA	111
Rattus-Col1a2-R	TGGCAGGCGAGATGGCTTAT	
Rattus-FN-F	ACGAGGAGGATGTGGCAGAG	205
Rattus-FN-R	GTGAATGAGTTGGCGGTGACA	
Rattus-α-sma-F	ACACGGCATCATCACCAACTG	253
Rattus-α-sma-R	TCCAGAGTCCAGCACAATACCA	
Rattus-Tmlhe-F	GCCATACCGATACCACCTACT	109
Rattus-Tmlhe-R	GCATAGAATCCATCCACCAACA	
Rattus-Gapdh-F	CAAGTTCAACGGCACAGTCAAG	123
Rattus-Gapdh-R	ACATACTCAGCACCAGCATCAC	

### Western blotting

The western blot analysis was conducted according to Taylor et al.’s method^20^ with a GAPDH monoclonal antibody (1:5,000, Abcam, ab181602) used as an internal control. The primary antibodies used were the TGF-β1 antibody (1:1,000, Affinity, AF1027), Smad3 antibody (1:1,000, Affinity, AF6362), α-SMA antibody (1:1,000, Abcam, ab32575), Col1a1 antibody (1:1,000, Abcam, ab34710), Col1a2 antibody (1:1,000, Abcam, ab34712) and FN antibody (1:1,000, Proteintech, 15613–1-AP), respectively.

### mRNA and lncRNA library preparation and sequencing

Ribosomal RNA in the samples was removed using the Ribo-Zero Golden kit (Epicentre, Illumina, USA), and cDNA libraries were prepared by a TruSeq total RNA sample preparation kit (Illumina, USA) according to the instructions. Fragments of 300–450 bp length were recovered using an agarose gel extraction kit (TIAGEN, China). All libraries were quantified using a 2100 Bioanalyzer and pooled at 1:1 at 2 nM for HiSeq 150 bp paired-end sequencing (Illumina, USA).

### Validation of miRNA target genes

HEK293 cells were seeded in cell culture plate and form 80% confluency after 24 h of culture. Hilymax-miRNA mimics complex and Hilymax-miRNA inhibitor complex were added to cell culture well as manufacturer’s instruction (Hilymax, Dojindo, Japan), and incubate at 37 °C in a CO_2_ incubator for 24 h. Cells were collected for RT-qPCR. The mimics and inhibitor were designed and synthesized by Generay Biotech Co, Ltd (Generay, China) (Table 2).

**Table 2:** The sequence of miRNA mimice and inhibitor.

	Sence	Antisence
miR-107 mimics	AGCAGCAUUGUACAGGGCUAUCA	AUAGCCCUGUACAAUGCUGCUUU
miR-107 inhibitor	UGAUAGCCCUGUACAAUGCUGCU	
miR-15b mimics	UAGCAGCACAUCAUGGUUUACA	UAAACCAUGAUGUGCUGCUAUU
miR-15b inhibitor	UGUAAACCAUGAUGUGCUGCUA	

### miRNA library preparation and sequencing

MicroRNA libraries were generated using the TruSeq Small RNA Library Preparation Kit (Illumina, USA) according to the manufacturer’s instructions. All libraries were purified and pooled at 1:1 at 2 nM and sequenced on an Illumina HiSeq platform using HiSeq 50SE single-end reads (Illumina, USA).

### LncRNA, mRNA and miRNA expression profiling

Sequence quality was estimated by using FastQC (v0.11.5)^21^. Adapters and low-quality reads were trimmed with Trimmomatic (v0.36)^22^. Reads with an average quality score of less than 20 after trimming were discarded. All the remaining qualified reads were mapped to the *Rattus norvegicus* genome (Rnor_5.0) for mRNA, lncRNA and microRNA analysis using HiSat2^23^. Then, we employed StringTie for gene quantification^24^.

Data preprocessing, Pearson correlation coefficient, and hierarchical clustering analysis (HCA) calculated by the “ward” method were performed in the statistical software R (www.r-project.org/) with its “base” function and “stat” packages. The TargetScan database (v7.2) and miRbase were employed for miRNA target gene prediction and further coexpression network computing^25^. Packages including “limma”, “edgeR”, “gplots”, and “ggplot2” were used to normalize the raw data and plot graphs.

### Differential expression analysis

The differential expression of genes was determined by calculating fold changes using the normalized value of each group, and statistical significance of the differentially expressed genes (DEGs) was presented by calculating a t test *p *value. Then, the significance of DEGs was determined according to the criteria of fold change larger than 1.5 or less than 0.67 and p-value less than 0.05.

### KEGG pathway enrichment analysis of differentially expressed genes

Upregulated genes and downregulated genes identified by comparing gene expression were used to query the KEGG pathway database to determine the biological function of these DEGs. Enriched pathways were determined by both significant Fisher’s exact test (*p *value < 0.05) and the involvement of at least 3 DEGs in a pathway. The pathway enrichment analysis was performed by using the “KEGG.db” and “KEGGprofile” packages in the R project (https://bioconductor.org/packages).

### CeRNA coexpression gene network analysis

Gene ceRNA (competing endogenous) and coexpression networks were built according to the normalized signal intensity of specific expressed genes. We defined the network adjacency between two genes, i and j, as a power of the Pearson correlation between the corresponding gene expression profiles. By computing the correlation coefficient of these genes, we obtained the gene–gene coexpression adjacency matrix, M (i,j), in which only genes with the strongest correlations (0.8 or greater) were selected to include in coexpression network graphs. Cytoscape (version 3.6.1) was used to plot the coexpression and ceRNA regulatory network^26^.

## Results

### Renal fibrosis rat modelling

**Figure 2:**
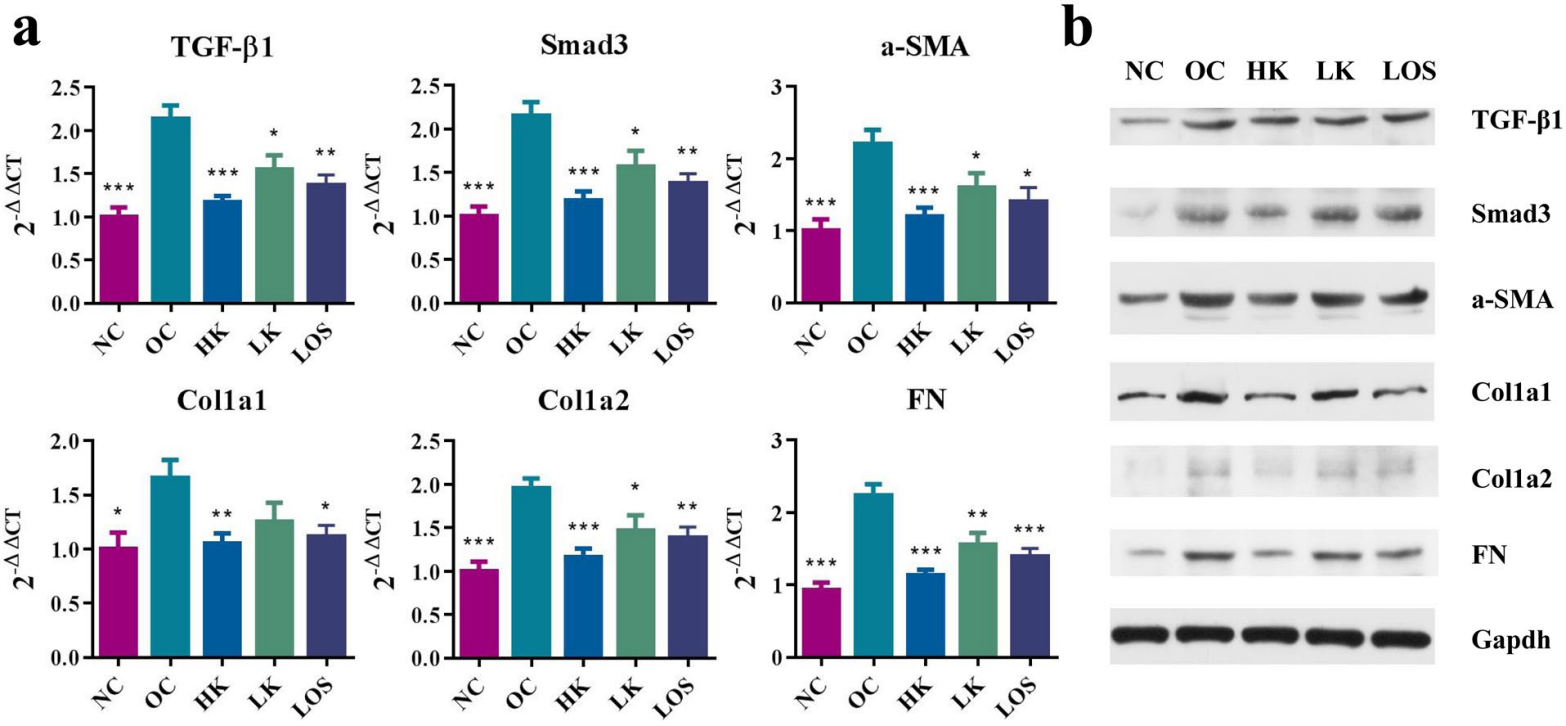
Marker validation in the RIF model. (**a**) The expression levels of the renal fibrosis-associated genes TGF-β1, FN, Smad3, Col1a1, Col1a2, and α-SMA were quantitated in each of the groups. All groups were compared with OC. *stands for *p* < 0.05, **stands for *p* < 0.01, and ***stands for *p* < 0.001. (**b**) The western blot analysis of TGF-β1, Smad3, a-SMA, FN, Col1a1 and Col1a2. Full-length blots are presented in Supplementary Fig. 1.

Compared with those in the OC group, the expression levels of BUN, Scr and 24 h urine protein decreased in the KXL treatment group and the LOS treatment group. The therapeutic effect increased as the KXL dose increased. The treatment effect in the HK group was better than that in the LOS group (Table 3). The same trend was also found in the RT-PCR detection and western blot analysis such as TGF-β1, Smad3, a-SMA, Col1a1, Col1a2 and FN (Fig. 2).

**Table 3:** Effect of KXL on serum creatinine (Scr), blood urea nitrogen (BUN) and 24-h urinary protein excretion.

Group	N	Scr (μmol/L)	BUN (mmol/L)	24-h urine protein (mg)
OC	5	23.90 ± 2.23^###^	40.63 ± 6.08^###^	50.34 ± 9.25^###^
LOS	5	20.91 ± 3.28***^,###^	27.53 ± 4.25*^,###^	36.75 ± 7.10**^,###^
LK	5	21.69 ± 2.21^###^	40.27 ± 5.96*^,###^	46.83 ± 7.94^###^
HK	5	16.63 ± 2.22***^,##^	25.38 ± 5.57***^,##^	34.30 ± 7.68***^,###^
NC	5	12.91 ± 3.00***	18.77 ± 2.35***	21.31 ± 2.24***

Values are means ± SD. *indicates groups compared with OC, and ^#^indicates groups compared with NC. *represent *p* < 0.05, **^,##^represent *p* < 0.01, ***^,###^represent *p* < 0.001.

**Figure 3:**
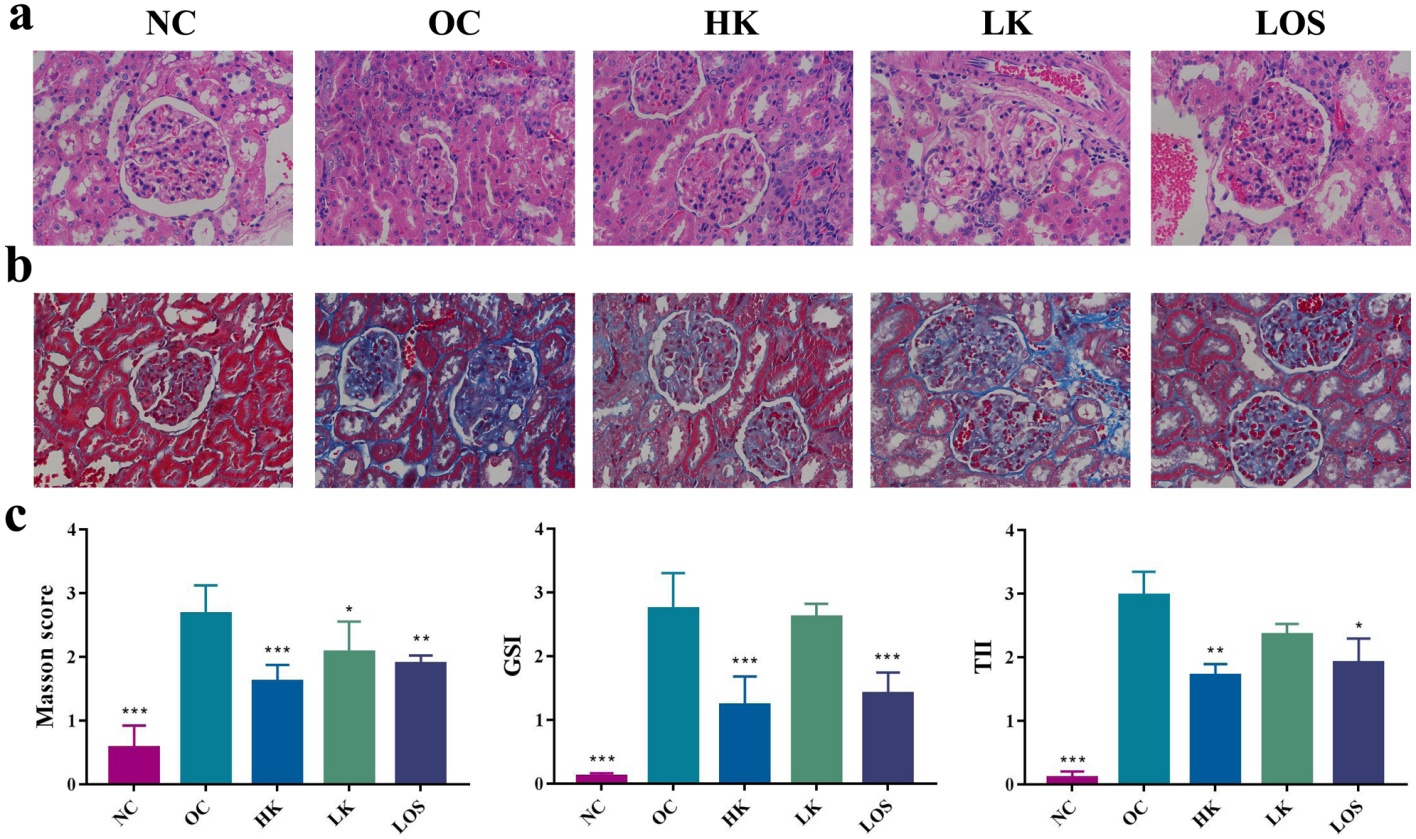
Pathology analyses of rat RIF. (**a**) HE staining (400 ×). (**b**) Masson staining (400 ×). (**c**) Masson score, GSI and TII of each group. All groups were compared with OC. * stands for P < 0.05, ** stands for P < 0.01, and *** stands for P < 0.001.

The GSI and TII scores of the HK group and LOS group were significantly decreased compared with those of the OC group (Fig. 3). The scores of the HK group were significantly decreased compared with those of the LK group. Masson staining showed that the NC group had green-stained collagen fibres only in the blood vessels and basement membrane, and the interstitial components were relatively small. In the OC group, collagen content increased, a large number of interstitial cells infiltrated, and green-stained collagen fibres increased significantly. The collagen fibre component and degree of interstitial cell infiltration of the HK group and LOS group were lower than those of the OC group.

### mRNA analysis

**Figure 4:**
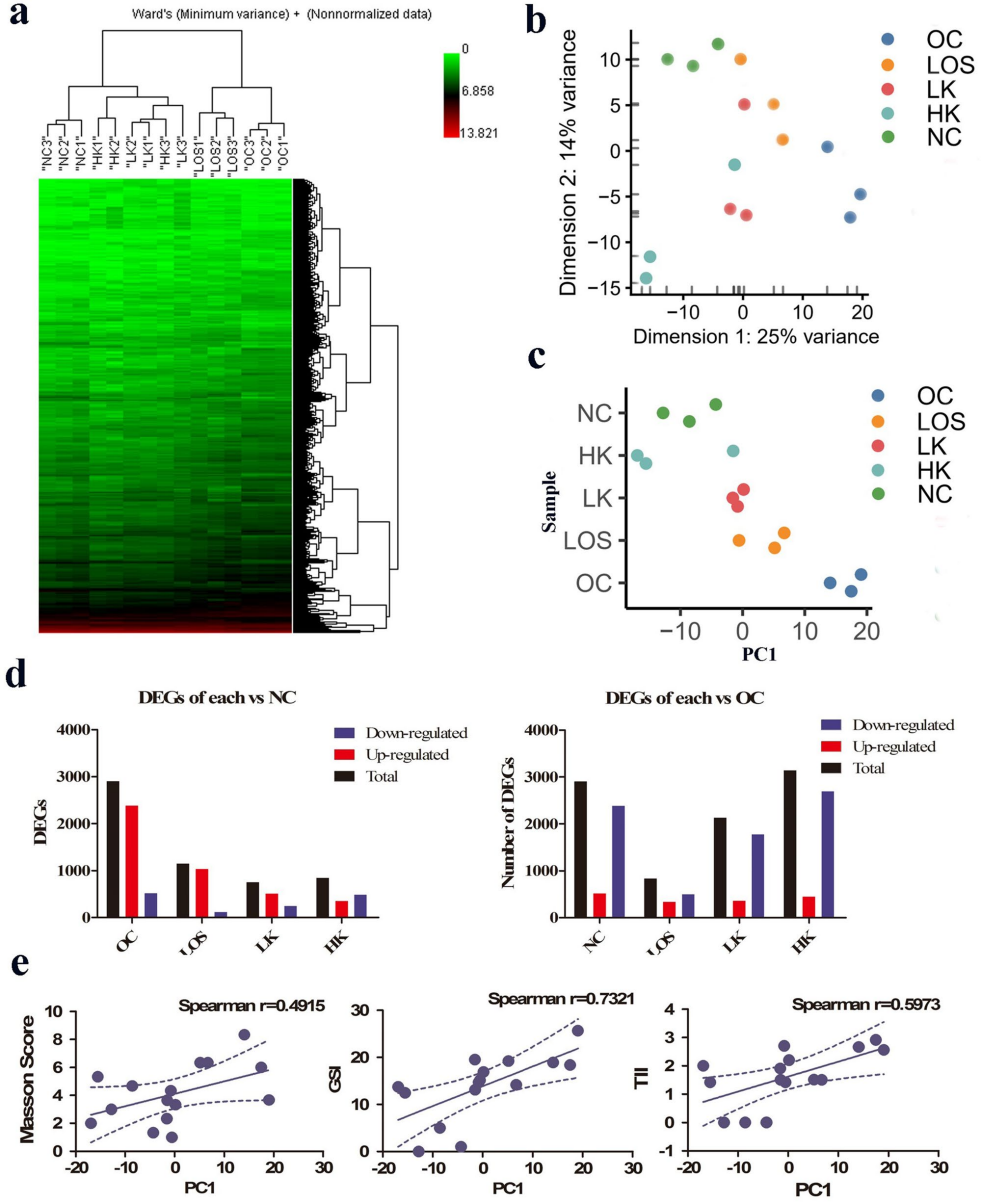
mRNA analysis. (**a**) Hierarchical clustering analysis of all samples. (**b**) T-SNE plot for all samples. (**c**) Eigenvalues by PC1 in each group. (**d**) Numbers of DEGs. (**e**) PC1 correlated with pathological assessments.

The T-SNE diagram shows an obvious distinction between the groups (Fig. 4a). By the HCA, we observed that samples from different experimental groups were well isolated and had good biological repeatability (Fig. 4b). Among the groups, NC and OC showed the biggest difference in terms of the number of DEGs, while the NC and HK treatment groups were the closest, indicating the therapeutic effect of KXL treatment (Fig. 4c−d). The correlation analysis between the pathological staining score and the PC1 dimension in Fig. 4e shows that the sequencing results were basically consistent with the pathological results.

**Figure 5:**
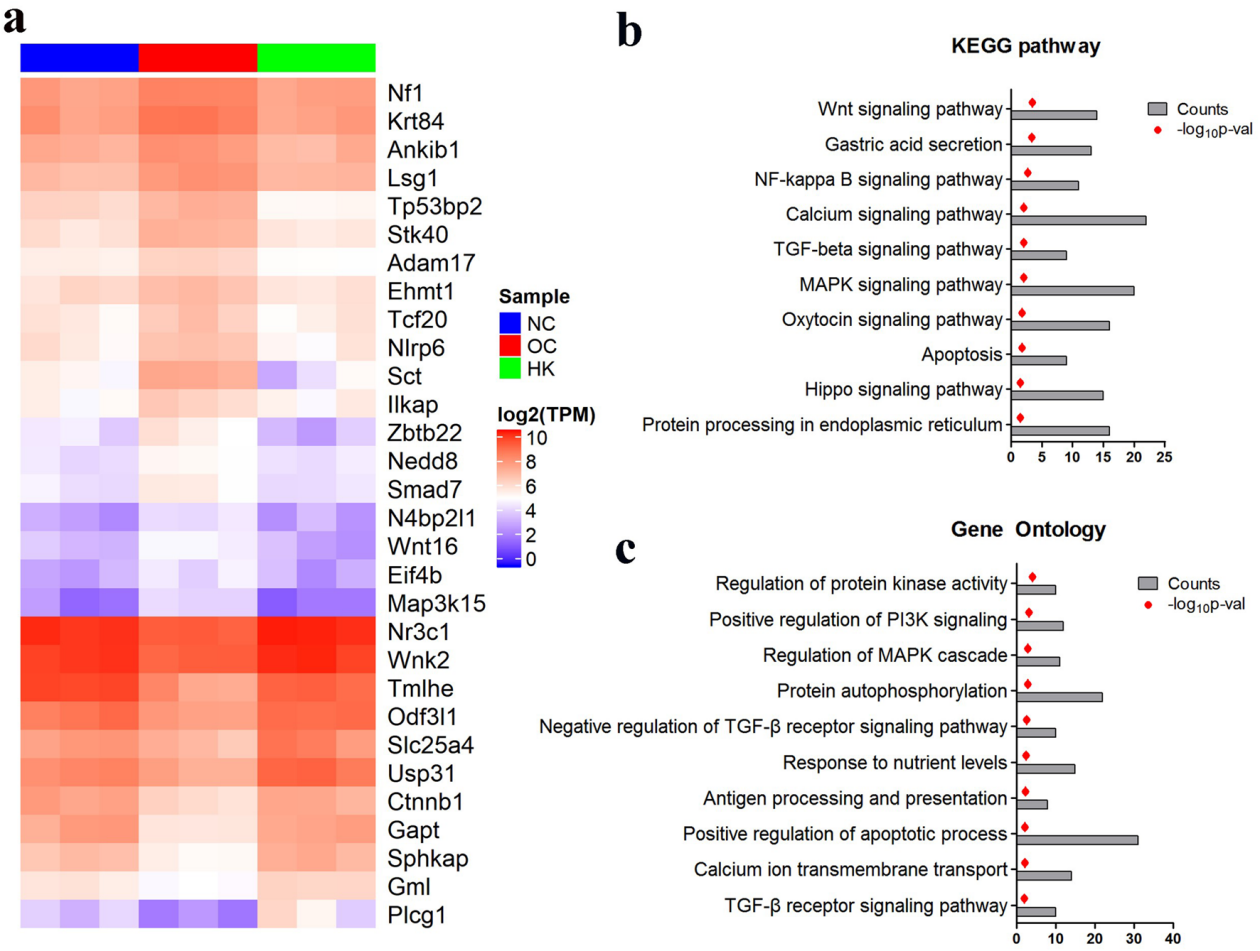
Functional analysis of the kidney transcriptome by KXL treatment. (**a**) Heatmap of high-dose KXL treatment-related genes. (**b**) KEGG pathway analysis of KXL treatment-related pathways. (**c**) GO analysis of KXL treatment-related functional annotations.

DEGs and heatmap analysis revealed that a group of genes, including Smad7, Wnt16 and Map3k15, were significantly altered by KXL (high dose) treatment, similar to the comparison of NC to OC to KXL (Fig. 5a). Furthermore, we performed KEGG pathway analysis and gene functional enrichment of DEGs. Pathway analysis confirmed that the fibrosis pathogenesis and anti-fibrosis processes were related to the Wnt signalling pathway (rno04310: WNT1, TBL1XR1, WNT7B, WNT16, PLCB4), TGF-β signalling pathway (rno04350: AMHR2, PPP2R1B, SP1, ROCK1, SMAD7, SMURF1, BMP7), NFκB pathway (rno04064: MAP3K7, DDX58, VCAM1, LAT, XIAP, RIPK1) and MAPK signalling pathways (rno04010: MEF2C, NF1, RELB, CACNB1, CACNB2, STK4, DAXX, TAB2, TGFB2) (Fig. 5b), while GO (Gene Ontology) analysis also showed that MAPK, PI3K and TGF-β were functionally enriched in the KXL treatment.

### Target gene and functional analysis of KXL treatment-related miRNAs

**Figure 6:**
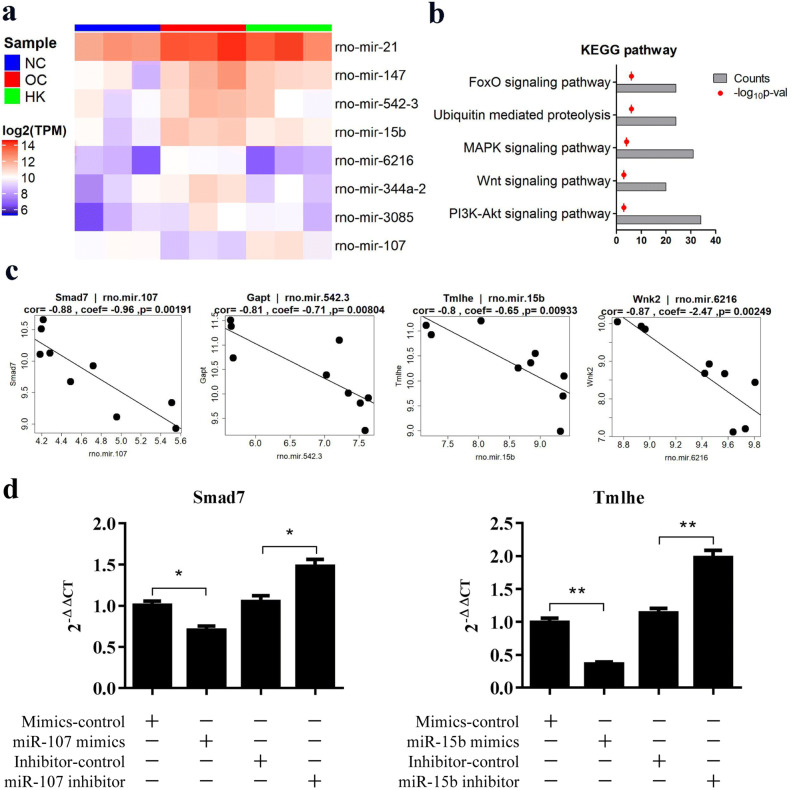
Target gene and functional analysis of KXL treatment-related miRNAs. (**a**) Heatmap of high-dose KXL treatment-related genes. (**b**) KEGG pathway analysis of KXL treatment-related miRNA target genes. (**c**) Negatively regulated miRNAs and their targets include Smad7, Gapt, Tmlhe and Wnk2. (**d**) Validation of miRNA target genes. *stands for *p* < 0.05 and **stands for *p* < 0.01.

DEG analysis of miRNA datasets showed that miR-15b, miR-21 and miR-6216 were upregulated in the fibrosis model and downregulated after KXL treatment. miR-107 was downregulated in the fibrotic kidneys and upregulated after drug treatment, which implied its important role in inhibiting renal fibrosis (Fig. 6a). Through database alignment, the target genes corresponding to the significantly dysregulated miRNAs were identified, and then the target genes were subjected to KEGG pathway analysis. As shown in Fig. 6b, the FoxO signalling pathway, MAPK signalling pathway and Wnt signalling pathway were significantly correlated with target genes underlying the roles of miRNAs in the regulation of fibrosis. We constructed a miRNA-mRNA coexpression network with DEGs of mRNAs and miRNAs, among which 94 genes, including Smad7, Map3k15, Gapt, Tmlhe, and Wnt16, satisfied the negative correlation coefficient lower than -0.8 criterion (Fig. 6c). RT-qPCR results showed that miR-107 mimics could suppress the expression of Smad7 in HEK293 cells. Moreover, inhibition of endogenous miR-107 by miR-107 inhibitor and resulted in the increase of expression of Smad7 in HEK293 cells. Thus, we conclude that miR-107 can down-regulate the expression of Smad7 in HEK293 cells. Similarly, we found that miR-15b can down-regulate Tmlhe expression in HEK293 cells (Fig. 6d).

### KXL treatment-related ceRNA co-network analysis

**Figure 7:**
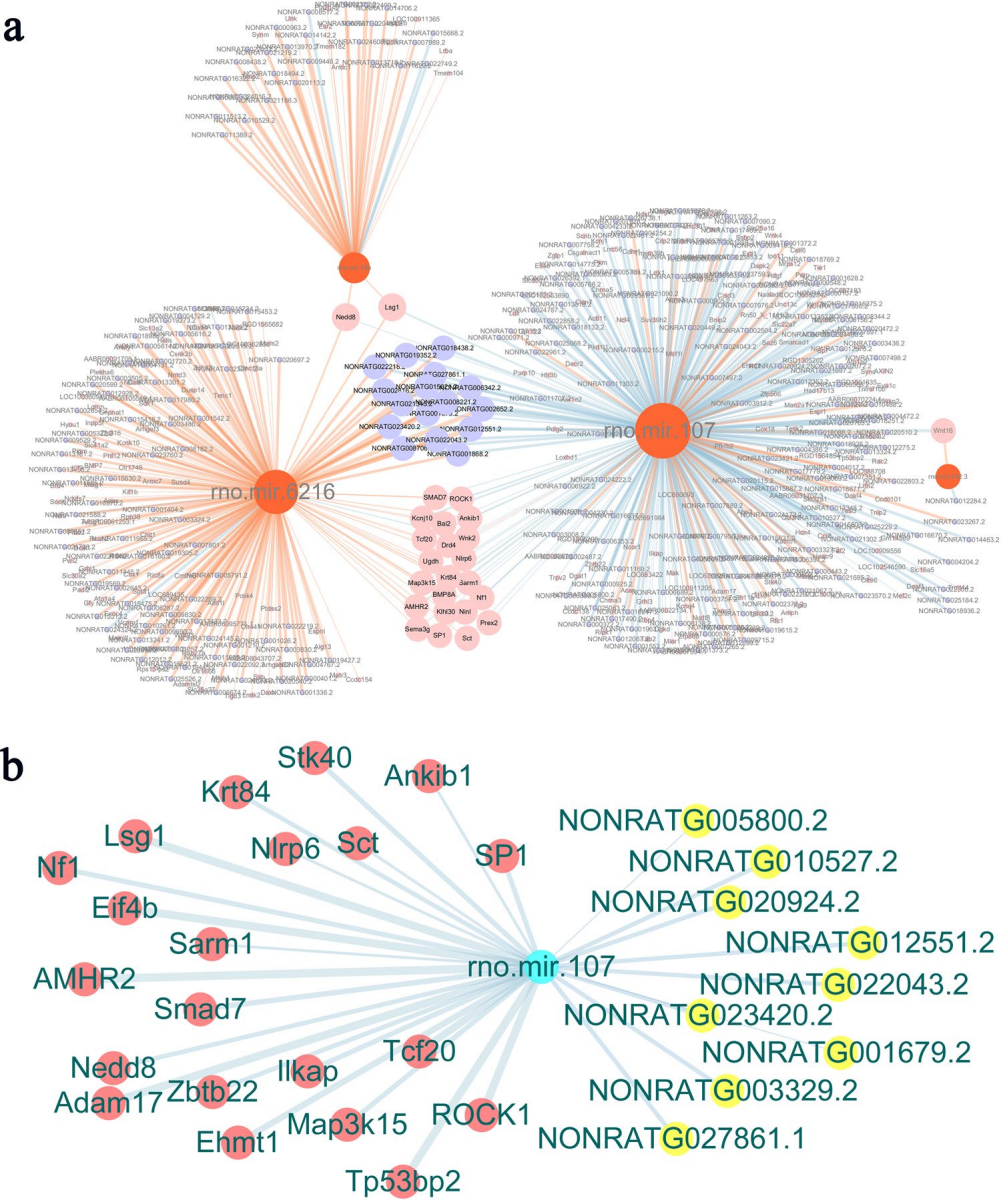
Treatment-based mRNA-lncRNA-miRNA ceRNA networks in the RIF kidney. (**a**) Three key miRNA-based ceRNA networks, based on miR-6216, miR-107 and miR-15b, were linked in the KXL treatment model and were enriched in the ceRNA network. (**b**) Several upregulated lncRNAs were enriched in the KXL treatment-related gene analysis, reducing the abundance of miR-107 and further enhancing the expression of Smad7, ROCK1, and Wnt16. Blue nodes represent mRNAs. Orange nodes represent miRNAs. Pink nodes represent lncRNAs. The edge breadth denotes the adjusted − log (*p *value), red denotes upregulation, and blue stands for downregulation.

In the lncRNA analysis, we found that a total of 1986 noncoding genes participated in gene-to-gene regulation. We established a potential ceRNA regulatory network based on the lncRNA-miRNA-mRNA expression pattern. In our analysis, miRNAs were considered to be regulated by lncRNAs to achieve inhibition or promotion of mRNAs. The ceRNA network was built based on three significantly enriched miRNAs, i.e., miR-6216, miR-107 and miR-15b (Fig. 7a). In terms of the fibrosis mechanism and therapeutic effect of KXL, we zoomed in on a miR-107-related network and found that several upregulated lncRNAs, such as NONRATG0020924.2, were enriched to reduce the abundance of miR-107 and further enhance the expression of anti-fibrosis-related genes, including Smad7, ROCK1, and Wnt16 (adjusted *p *value < 0.05) (Fig. 7b).

## Discussion

In this study, we showed effective anti-fibrotic activity in the renal interstitial fibrosis rat model using KXL, a traditional Chinese medicine. However, the main concern in promoting the ancient but effective therapies is the explanation of mechanisms of action of the formulas^27^.

The main mechanisms of RIF and therapeutic effect of the KXL herbal formula in our study were explained by multi-omics sequencing, which revealed that more than 1,800 signature genes including Smad7, Wnt16 and Map3k15 were upregulated. This further triggered the downstream activation of the Wnt, TGF-β and MAPK pathways. Surenden et al.^28^ reported Wnt family gene involvement in a unilateral ureteral obstruction model (UUO) in mice. A number of studies^29,30^ have shown that the Wnt signalling pathway does not independently exist and regulate renal fibrosis but interacts with a variety of factors and other signalling pathways. Wnt/β-catenin interacts with the RTK/Ras/MAPK pathway and the PI3K/ILK/PKB pathway to promote EMT^31–33^.

In the analyses of miRNAs in RIF, miR-15b, miR-107, miR-21 and miR-6216 were shown to play critical roles in the pathogenesis and therapeutics of renal fibrosis. Dysregulation of miR-107 was reported to inhibit the migration and invasion of cells by upregulation of PTEN and inhibition of PI3K signalling^34^. Overexpression of miR-107 regulates the expression of VEGF^35^. The EMT process in RIF can also be inhibited by the blockage of the miR-21/PTEN/PI3K pathway^36^. The miR-15 family was reported as a novel regulator in cardiac fibrosis acting via inhibition of the TGF-β pathway^37^.

Angiotensin II (ANG II) is a major pathogenic factor of renal fibrosis in chronic renal injury. Losartan is an antagonist of ANG II that binds to the AT1 receptor. Losartan was shown to have renoprotective effects on rats with 5/6 subtotal nephrectomy^38^. The results by He et al. indicated that losartan attenuated renal fibrosis and tubular cell apoptosis in a UUO rat model by blocking STAT3 phosphorylation^39^. Our pathological assessment results showed that high dose of KXL had a great benefit than losartan in RIF treatment. The sequencing results further demonstrated and explained the mechanisms by the number of DEGs and enriched pathways.

Based on lncRNA, mRNA and miRNA analysis, we established a potential ceRNA regulatory network. Several upregulated lncRNAs, including NONRATG0020924.2, were enriched in kidney fibrosis. The anti-fibrotic effect could also be achieved by disturbing this network to reduce the expression of fibrotic factors such as the Smad and Wnt genes. For the first time, we elucidated the correlations between differentially expressed mRNAs and lncRNAs and delineated the functional landscapes of the mRNA-miRNA-lncRNA ceRNA network in the pathogenesis and therapeutics following anti-fibrotic treatment with KXL. The TGF-β/Smad network plays an important role in renal fibrosis, and Smad7 has been shown to be a therapeutic agent for renal fibrosis in various models of kidney diseases^40^. CeRNA networks of lncRNA/miRNA/mRNA interactions show a more universal and crucial regulatory role in fibrosis^41,42^. These regulatory network studies drew similar conclusions as ours.

In recent years, TCMs have been rapidly adopted not only in China but also in many countries in the rest of the world. In this study, we established a kidney RIF treatment model by TCM, and the therapeutic effects of KXL were explained by multi-omics approaches. These results supported the clinical use of KXL in practice.

## Supplementary information

Supplementary file1 (PDF 724 kb)

## Data Availability

The datasets generated during and analysed during the current study are available from the corresponding author on reasonable request.
