# Supplementary material for: Transcriptomic analysis of the mechanisms of alleviating renal interstitial fibrosis using the traditional Chinese medicine Kangxianling in a rat model
Source: Sci Rep. 2020 Jun 30;10:10682. doi: 10.1038/s41598-020-67690-3 (PMC7327068; doi:10.1038/s41598-020-67690-3)

Transcriptomic analysis of the mechanisms of alleviating renal interstitial fibrosis using the traditional Chinese medicine Kangxianling in a rat model

Yufeng Jiang, Yaohan Zhu, Timing Zhen, Jie Li, Kaichen Xing, Liqun He, Sibo Zhu.

Supplementary Figure 1. Original blots for Figure 2b.

We indicate the contours of the membranes by marked frames and the image which is shown in Figure 2b.

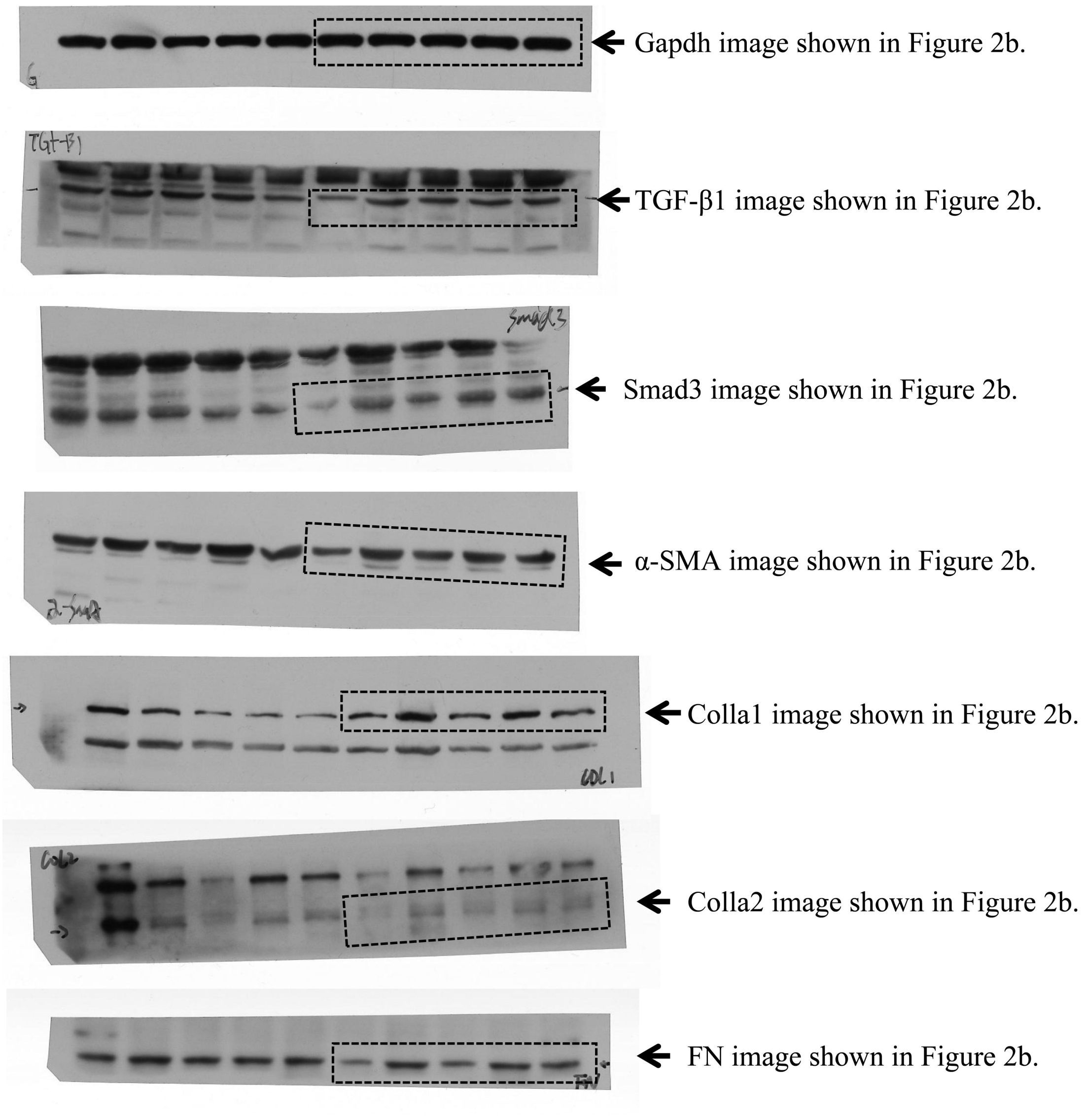

Supplement: Supplementary file 1 — Supplementary file1 (PDF 724 kb) [file 41598_2020_67690_MOESM1_ESM.pdf]
